# Supplementary material for: FERN – a Java framework for stochastic simulation and evaluation of reaction networks
Source: BMC Bioinformatics. 2008 Aug 29;9:356. doi: 10.1186/1471-2105-9-356 (PMC2553347; doi:10.1186/1471-2105-9-356)
Supplement: Additional file 1 — FERN distribution, Version 1.3. This archive contains the FERN source code and binaries as well as documentation and example models in FernML and SBML. [file 1471-2105-9-356-S1.zip › fern/doc/javadoc/fern/network/package-summary.html]

fern.network


---


|  |  |  |
| --- | --- | --- |
| **Overview** \| **Package** \| Class \| **Use** \| **Tree** \| **Deprecated** \| **Index** \| **Help** | |  |
| **PREV PACKAGE**   **NEXT PACKAGE** | **FRAMES**    **NO FRAMES**     **All Classes** |  |


---

## Package fern.network

Provides general classes and interfaces for storing network data.

**See:**
  
          **Description**

| **Interface Summary** | |
| --- | --- |
| **AnnotationManager** | AnnotationManager objects are the places, where static (or quasi-static) properties of a network are stored. |
| **ComplexDependenciesPropensityCalculator** | In a SBML network, the propensities of reaction are calculated by using the kineticLaw tag, which contains a MathML expression. |
| **Network** | The central interface of the whole framework. |
| **PropensityCalculator** | A `PropensityCalculator` is the way, a `Network` tells a `Simulator` how to calculate propensities for the reactions. |

| **Class Summary** | |
| --- | --- |
| **AbstractKineticConstantPropensityCalculator** | Base implementation of a `PropensityCalculator`. |
| **AbstractNetworkImpl** | Base implementation for the `Network` interface. |
| **AmountManager** | The `AmountManager` is one of the most important connections between a `Network` and a `Simulator`. |
| **AnnotationManagerImpl** | Base implementation of the `AnnotationManager` interface. |
| **ArrayKineticConstantPropensityCalculator** | Implementation of an `AbstractKineticConstantPropensityCalculator` which uses an array to store the constants for each reaction. |
| **NetworkLoader** |  |

| **Exception Summary** | |
| --- | --- |
| **FeatureNotSupportedException** |  |

## Package fern.network Description

Provides general classes and interfaces for storing network data.

---


|  |  |  |
| --- | --- | --- |
| **Overview** \| **Package** \| Class \| **Use** \| **Tree** \| **Deprecated** \| **Index** \| **Help** | |  |
| **PREV PACKAGE**   **NEXT PACKAGE** | **FRAMES**    **NO FRAMES**     **All Classes** |  |


---
